# A Feasible ^1^H‐NMR Quantification Method of Indicator Compounds in Microwave‐Processed *Reynoutria multiflora* Radix and Its Decoction

**DOI:** 10.1155/ianc/6092060

**Published:** 2026-10-18

**Authors:** Hao-Ze Li, Hong Nhung Phan, Zih-Hong Jhang, Guo-Fen Chen, Mei-Lin Yang, Tian-Shung Wu, Ping-Chung Kuo

**Affiliations:** ^1^ School of Pharmacy College of Medicine National Cheng Kung University Tainan 70101 Taiwan ncku.edu.tw; ^2^ Department of Chemistry National Chung-Hsing University Taichung 402 Taiwan nchu.edu.tw

**Keywords:** 2,3,5,4′-tetrahydroxystilbene-2-O-*β*-D-glucopyranoside (THSG), anthraquinone, quantitative nuclear magnetic resonance spectroscopy (qNMR), *Reynoutria multiflora*

## Abstract

*Reynoutria multiflora* (Thunb.) Moldenke is a commonly used herbal drug in traditional Chinese medicine. Prior literature has indicated that *R. multiflora* possesses broadband pharmacological activities. According to Taiwan Herbal Pharmacopeia (IV) and previous reports, the dominant components of *R. multiflora* were 2,3,5,4′‐tetrahydroxystilbene‐2‐O‐*β*‐D‐glucopyranoside (THSG) (**1**) and free anthraquinones such as emodin (**2**), physcion (**3**), and rhein (**4**), which played pivotal roles in the pharmacological effects of herbs. However, the standard analytic method with HPLC‐UV took much time to prepare samples and calibration curves based on the standard substances. In this study, a rapid quantitative ^1^H‐NMR analytic method has been developed, which can quantify the indicator compounds in *R. multiflora* samples. The method validation data demonstrated the good accuracy and precision of this qNMR method. The feasible analytical method can monitor the contents of indicator compounds in both raw and processed herbs. In addition, this method could be applied to analyze the indicator compounds in the decoction Qi Bao Mei Ran Dan successfully. These results all supported the proposed method as a useful tool for quality control of *R. multiflora* herbal materials and decoctions. With further refinement, this method could serve as a reference for future standard methods in pharmacopoeias.

## 1. Introduction

High‐resolution nuclear magnetic resonance (NMR) spectroscopy has been employed to elucidate the structures of compounds derived from synthetic or natural products for several decades. Recently, this technique was also applied to the quality assessment of *R. japonica* and *R. multiflora*, indicating that ^1^H‐NMR analysis can obtain their metabolic fingerprints to distinguish between the two species [1]. Quantitative NMR (qNMR) was a novel application of NMR for quantitative analysis. Recently, qNMR has been developed and is regarded as a potential tool for the quality control of natural products [2–6], pharmaceuticals [7–9], clinical diagnosis, and monitoring of treatment [10]. Compared to the classical analytic methods, qNMR has several advantages. First, the sample preparation was simple and rapid without complex pretreatment steps. Second, it used the internal standard (IS) method without preparing calibration curves based on the standard substances. Third, no advanced isolation was required before monitoring multiple compounds in herbal samples. Therefore, an increasing number of research articles related to qNMR have been published recently.

*Reynoutria multiflora* Radix, a famous herb in traditional Chinese medicine, is known for its extensive therapeutic effects. According to the 4th edition of Taiwan Herbal Pharmacopeia, this herb is the dried root tuber of *Reynoutria multiflora* (Thunb.) Moldenke, a species of herbaceous flowering plant belonging to the *Reynoutria* genus and the Polygonaceae family [11]. The herbal usage form of *R. multiflora* could be divided into two states, which possess different pharmacological properties. First was the raw state, *Reynoutria multiflora* Radix, used to treat constipation and prevent malaria. The other was the processed state named *Reynoutria multiflora* Radix Preparata, mainly used for nourishing the liver and kidney, as a tonic drug, blackening hair, and eliminating dampness [12, 13]. They were used as common herbs for treating various diseases and as dietary supplements. The major chemical constituents isolated and identified from *R. multiflora* were stilbene glycosides and anthraquinones. According to previous quantitative reports, the content of 2,3,5,4′‐tetrahydroxystilbene‐2‐O‐*β*‐D‐glucopyranoside (THSG) (**1**) (Figure 1) was the most abundant, and THSG also exhibited antiaging, anti‐inflammatory, cardiac‐protective, and hepatoprotective bioactivities [13, 14]. Emodin (**2**), physcion (**3**), and rhein (**4**) (Figure 1) belonging to anthraquinones were other notable ingredients in *R. multiflora*, which also possessed antioxidant, antitumor, antiviral, and hepatoprotective functions [15, 16]. Since these components were the dominant principles and showed various biological activities, they played pivotal roles in the pharmacological effects of *R. multiflora*.

**FIGURE 1:**
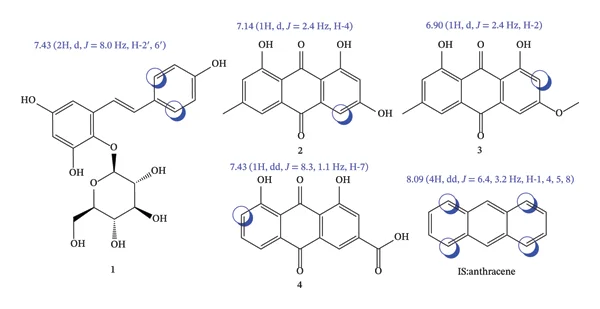
The structures of compounds **1**–**4** and internal standard.

The standard analytic method for *R. multiflora* provided in Taiwan herbal pharmacopeia examined the prepared sample solutions and standard solutions with HPLC‐UV [11]. It had also been reported to utilize UPLC to quantify the contents of THSG (**1**), emodin (**2**), and physcion (**3**) simultaneously [17, 18]. Nevertheless, these procedures were tedious and required a significant amount of time and solvent. Moreover, only THSG (**1**) could be clearly identified in this method (Figure S1). Hence, in the present study, we aimed to explore a feasible qNMR method to quantify the significant compounds such as THSG (**1**), emodin (**2**), physcion (**3**), and rhein (**4**) in plant materials. This qNMR method would be utilized to monitor the contents of these significant compounds in raw and processed samples of *R. multiflora*, as well as the decoction of *R. multiflora*.

## 2. Chemical Substances

All the chemicals, unless specifically indicated otherwise, were purchased from Merck KGaA (Darmstadt, Germany). Anthracene (97.0%) and DMSO‐*d*_6_ (99.9%) were obtained from Sigma‐Aldrich (St. Louis, MO, USA). The ultrapure water was prepared with the Milli‐Q water purification system (Millipore, Bedford, MA, USA). The reference compound **1** was obtained from Macklin (Shanghai, China), **2** and **3** were purchased from Sigma‐Aldrich, and **4** was bought from LGC Ltd. (Toronto, Canada).

## 3. Experimental Part

### 3.1. Plant Materials and Commercial Products

Herbal materials of *R. multiflora* (Thunb.) Moldenke (NCKU_Kuo_2021005∼2021012) were collected from the various traditional Chinese medicine market suppliers in Taiwan. The commercial products of decoction Qi Bao Mei Ran Dan were purchased from three different traditional Chinese medicine pharmaceutical companies in Taiwan. All the voucher specimens and commercial products of decoction had been deposited in the herbarium of the School of Pharmacy, National Cheng Kung University, Tainan, Taiwan. The detailed information on sample characteristics is listed in the Supporting Information (Table S1).

### 3.2. Microwave Processing

A home‐use microwave oven (Sampo, RE‐1002SM, 30 L) capable of generating 900‐W power at 2450 MHz was used for processing. A serving of 20.0 g of raw *R. multiflora* material was weighed and processed in the microwave oven. Power (270–540 W), processing time (1–2 min), and repetition were adjusted to produce the processed materials.

### 3.3. Extraction

Granular powdered samples of *R. multiflora* (about 0.5 g) were immersed in 10 mL of distilled methanol, extracted by an ultrasonicator for 10 min, and then centrifuged at 3000 rpm for 7 min. The supernatant part was collected and concentrated with a rotary evaporator, and the above steps were repeated three times. The extractants collected were lyophilized to produce the *R. multiflora* extracts.

### 3.4. Partition

The methanol extracts were divided into chloroform and water layers by liquid–liquid partition between chloroform and water. The water layer was lyophilized and then re‐dissolved in distilled methanol, and the solution was diluted to 25 mL in a volumetric flask (solution A). The solvent from the chloroform layer was evaporated using a rotary evaporator, and the residue was dissolved and diluted to 25 mL with distilled acetone in a volumetric flask (solution B). Anthracene (10 mg) was dissolved with distilled acetone in a 10‐mL volumetric flask to produce 1 mg/mL of IS solution.

### 3.5. Sample Preparation

Aliquots of solution A (2.5 mL) and solution B (5 mL) were each independently mixed with 0.5 mL of IS solution to prepare two separate sample solutions (A + IS and B + IS). Each mixture was transferred into a vial, evaporated to dryness, and subsequently lyophilized before qNMR analysis. The precise weights of the added samples and ISs have been recorded in the Supporting Information (Table S2).

### 3.6. ^1^H qNMR Acquisition

Quantitative ^1^H‐NMR analyses were performed on a Bruker AV‐400 (Bruker, Billerica, MA, USA) 400 MHz NMR spectrometer, with the solvent peak of DMSO‐*d*_6_ at 2.50 ppm to calibrate the chemical shift of our spectra. Chemical shifts were reported in parts per million (ppm, *δ*). After lyophilization, the residues of water and chloroform layer samples were dissolved in 0.8 mL of DMSO‐*d*_6_, and 0.6 mL of the dissolved samples was transferred into a standard 5‐mm NMR tube. To ensure accurate quantification, a sufficient relaxation delay must be guaranteed. It is generally recommended to set the relaxation delay times to *5* × *T*_1_ to allow the nuclei to fully relax. The prepared samples were analyzed using a standard NMR 1D pulse sequence with the following parameters. A total of 100 scans were recorded in 0.187 Hz/point; spectra width, 3600 Hz; a 90° pulse was used to obtain the maximum sensitivity; relaxation delay, 20 s; acquisition time, 2.56 s. For the quantitative analysis, the peak area was used, and the start and end points of the integration of each peak were selected manually. All *R. multiflora* samples were analyzed in triplicate. The content results were calculated using the following equations, expressed as molar concentrations, and subsequently converted to mg/g for comparison with the pharmacopeial criteria.

(1) The content of indicator compound mM=A×C×DB×E.

A: Peak area of each indicator compound.

B: Peak area of anthracene.

C: The concentration of anthracene (mol/L).

D: The number of contributing nuclei for the selected peak of anthracene.

E: The number of contributing nuclei for the selected peak of the indicator compounds.

(2) The content of indicator compound %=A×C×D×GB×E×F×10.

A: Peak area of each indicator compound.

B: Peak area of anthracene.

C: The concentration of anthracene (mol/L).

D: The number of contributing nuclei for the selected peak of anthracene.

E: The number of contributing nuclei for the selected peak of the indicator compounds.

F: The weight of powdered *R. multiflora* samples for extract (g).

G: Molecular weight of each indicator compound.

The signals of THSG (**1**) at *δ* 7.43 (2H, *d*, *J* = 8.0 Hz, H‐2′, 6′), emodin (**2**) at *δ* 7.14 (1H, *d*, *J* = 2.4 Hz, H‐4), physcion (**3**) at *δ* 6.90 (1H, *d*, *J* = 2.4 Hz, H‐2), and rhein (**4**) at *δ* 7.43 (1H, dd, *J* = 8.3, 1.1 Hz, H‐7) were selected to quantify their levels. The anthracene peak at *δ* 8.09 (4H, dd, *J* = 6.4, 3.2 Hz, H‐1, 4, 5, 8) was selected for calculating the content of the indicator compounds.

### 3.7. Characterization of Indicator Compounds by 2D NMR Analysis

The solutions A and B were subjected to 2D NMR analyses, including gHSQCAD, gHMBCAD, and HSQC‐TOCSY experiments, using an Agilent DD2 600 MHz NMR spectrometer (National Chung‐Hsing University, Taichung, Taiwan). Detailed acquisition parameters for each 2D NMR experiment are provided in the Supporting Information (Table S3). JEOL Delta V5 software was used for data processing.

### 3.8. Method Validation

To ensure the suitability of the analytical working range of the developed method, the limit of detection (LOD) and limit of quantification (LOQ) were evaluated. The LODs and LOQs of the indicator compounds THSG (**1**), emodin (**2**), physcion (**3**), and rhein (**4**) in this qNMR method (100 scans) were determined by serial dilution of standard solutions. Prepare standard solutions at a concentration of 0.8 mg/mL, and then dilute to 0.4, 0.2, 0.1, 0.05, 0.025, and 0.0125 mg/mL. The LOD and LOQ values were estimated based on signal‐to‐noise (S/N) ratios of 3 and 10 from the recorded NMR spectra, in accordance with the ICH analytical guidelines. For method validation, recovery tests were performed at three concentration levels (low, medium, and high). Standard solutions at known concentrations were prepared in 10‐mL volumetric flasks. An aliquot of 5 mL of each standard solution was mixed with the IS solution (1 mg/mL) and subsequently lyophilized prior to measurement, following the same procedure as that used for the ^1^H NMR analysis. Recovery (%) was calculated as the ratio of the measured content to the amount of standard added. Precision was evaluated in terms of intra‐ and interday variations based on concentrations calculated from the quantitative equation. The steps above were performed in triplicate at low, medium, and high concentration levels and repeated on different days.

## 4. Results

### 4.1. Development of the qNMR Method for Analysis of the Indicator Compounds

After trying several deuterated solvents, DMSO‐*d*_6_ has been adopted as the solvent for NMR analysis. As a versatile organic solvent with excellent dissolving capacity, it ensures the complete dissolution of all components. The methanol extracts of *R. multiflora* samples were partitioned prior to conducting qNMR analysis. Among the indicator compounds in *R. multiflora* samples, THSG (**1**) exhibited higher polarity due to its glucose‐containing structure, which prefers to dissolve in higher polarity solvents, such as water. In contrast, anthraquinones such as emodin (**2**) and physcion (**3**) showed lower polarity and significant lipophilicity. As a result, the methanol extracts were divided into chloroform and water layers by liquid–liquid partition. In this study, anthracene was selected as the IS for the following reasons. First, anthracene exhibits a simple aromatic proton signal pattern, enabling accurate integration and minimal overlap with most target analyte signals. Second, it is readily soluble in common organic NMR solvents (e.g. DMSO‐*d*_6_) and is chemically inert toward the analytes, thereby preserving sample integrity. Third, as a stable polycyclic aromatic hydrocarbon, anthracene is commercially available in high purity and serves as a reliable reference for quantitative analysis. In the preliminary experiments, different concentrations of the IS were evaluated to optimize analytical performance. To ensure consistency across all measurements, a fixed amount of IS (1 mg/mL, 0.5 mL) was ultimately selected, which was found to be suitable for most samples analyzed in this study. The peaks of standard compounds and anthracene did not overlap, and the integral values of anthracene remained constant for more than 48 h. Hence, the water and chloroform fractions were respectively blended with the anthracene IS, and their qNMR spectra were recorded in DMSO‐*d*_6_. The chemical shifts of the indicator compounds were assigned by comparison with those reported in the literature [19, 20], and our spectral data are provided in the supporting information (Figures S2–S5).

Commercial *R. multiflora* samples were conducted with the procedures for qNMR analysis, and the afforded ^1^H‐NMR spectra are shown in Figure 2A–D. The spectrum of the water layer (Figure 2A,B) indicated THSG (**1**) as the major ingredient. However, the baseline around the signals at *δ* 6.17, 6.54, and 6.73 ppm displayed fluctuations, and the integrals of peak areas may result in bias. Hence, the peak at *δ* 7.43 ppm was selected as the target peak for calculating the content of THSG (**1**). Comparatively, in the ^1^H‐NMR spectra of the chloroform layer (Figure 2C,D), anthraquinones were the dominant components, except for rhein (**4**), which was not detected. Moreover, the spectrum showed that the peak of emodin (**2**) at *δ* 7.53 ppm and the peak of physcion (**3**) at *δ* 7.55 ppm would overlap with the signal of anthracene, and the peaks at *δ* 7.20–7.23 ppm for emodin (**2**) and physcion (**3**) also exhibited overlap. Therefore, the signals of emodin (**2**) at *δ* 7.14 and physcion (**3**) at *δ* 6.90 ppm were selected to quantify their levels. As discussed above, this qNMR method could locate the signals of THSG (**1**), emodin (**2**), and physcion (**3**), excluding rhein (**4**) due to its limited quantity, and the suitable peaks were selected to determine the concentrations of indicator compounds.

**FIGURE 2:**
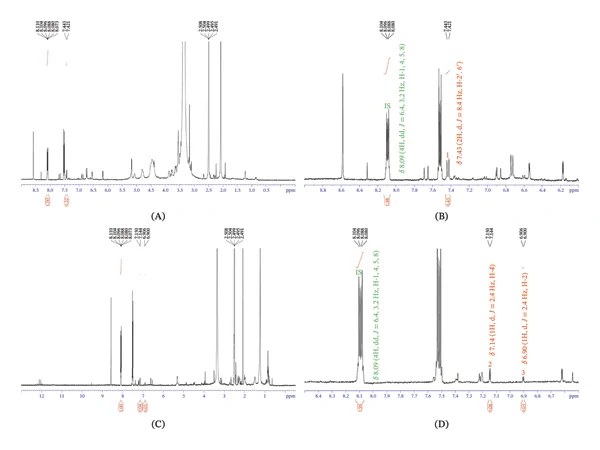
(A) ^1^H‐NMR spectrum of solution A + IS of sample 8 (DMSO‐*d*_6_, 400 MHz, 100 scans, IS: anthracene). (B) Expanded 6–9 ppm region ^1^H‐NMR spectrum of solution A + IS of sample 8 (DMSO‐*d*_6_, 400 MHz, 100 scans, IS: anthracene). (C) ^1^H‐NMR spectrum of solution B + IS of sample 8 (DMSO‐*d*_6_, 400 MHz, 100 scans, IS: anthracene). (D) Expanded 6.5–8.5 ppm region ^1^H‐NMR spectrum of solution B + IS of sample 8 (DMSO‐*d*_6_, 400 MHz, 100 scans, IS: anthracene).

### 4.2. Characterization of Indicator Compounds by 2D NMR Analysis

To effectively overcome severe spectral overlap and complex matrix interference in herbal extracts, the advanced two‐dimensional NMR techniques, specifically gHSQCAD and gHMBCAD were employed. By leveraging gradient selection and adiabatic decoupling, these methods significantly improve signal resolution and S/N ratio. While gHSQCAD identified direct ^1^H‐^13^C connectivity, gHMBCAD elucidated long‐range correlations (2_*J*_,  3_*J*_) to bridge quaternary carbons, enabling rapid structural elucidation of crude extracts without prior purification. For congested regions like carbohydrate domains, HSQC‐TOCSY functioned as a crucial diagnostic tool. In the total correlation spectroscopy (TOCSY), correlations between all protons within a given spin system were detected as long as there are couplings between every intervening hydrogen. It is very useful to identify molecules with complicated proton signals, such as amino acids and sugars. By resolving homonuclear (^1^H‐^1^H) spin systems across the ^13^C dimension, this hybrid technique effectively segregated overlapping proton signals, allowing for the unambiguous assignment of individual structural units. Through gHSQCAD, gHMBCAD, and HSQC‐TOCSY analyses (Figure 3A–C), the accurate and rapid assignments of THSG (**1**) and emodin (**2**) were achieved directly from *R. multiflora* samples without the need for cumbersome purification.

**FIGURE 3:**
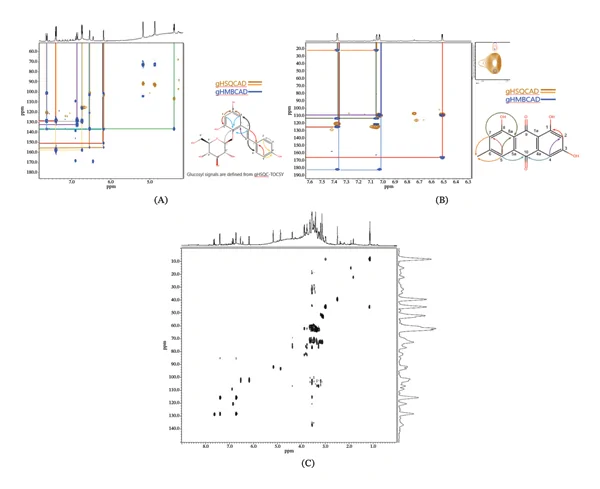
(A) gHSQCAD and gHMBCAD spectrum of solution A (DMSO‐*d*_6_, 600 MHz, 32 scans). (B) gHSQCAD and gHMBCAD spectrum of solution B (DMSO‐*d*_6_, 600 MHz, 32 scans). (C) gHSQC‐TOCSY spectrum of solution A (DMSO‐*d*_6_, 600 MHz, 32 scans).

### 4.3. Method Validation of qNMR Analysis

For method validation, the LODs and LOQs for the indicator compounds under the present NMR analytical condition were determined by serial dilution and recording the NMR spectra to examine the S/N ratio. Accordingly, the LODs and LOQs of THSG (**1**), emodin (**2**), physcion (**3**), and rhein (**4**) were 0.12 and 0.49 mM, 0.19 and 0.37 mM, 0.09 and 0.35 mM, and 0.09 and 0.19 mM, respectively. The ^1^H‐NMR spectra of LODs and LOQs for these indicator compounds are shown in Figures S6–S21. The selectivity of the qNMR method was evaluated by confirming the identities of the signals selected for quantification and assessing potential spectral interference from other components in the extract. The assignments of the selected quantitative signals were confirmed using HSQC and TOCSY experiments (Figure 3A–C). In addition, the corresponding regions of the ^1^H NMR spectra were examined for potential signal overlap. The selected signals were sufficiently resolved and showed no significant interference from other constituents of the extract, demonstrating adequate selectivity for quantitative analysis. Recovery tests were selected to examine the accuracy of the method, in which the known concentrations of indicator compounds were recorded, and the recovery percentages were calculated by dividing the measured contents by the contents of added standards. The analysis was performed in triplicate at low, medium, and high concentration levels (presented by LQC, MQC, and HQC) on different days to evaluate the precision in terms of intra‐ and interday variations based on concentrations calculated from the quantitative equation. According to the validation results, all groups exhibited good recovery. In terms of precision, only the THSG (**1**) LQC and Rhein (**4**) LQC groups showed an RSD > 5%. However, these ranges remained within acceptable limits in ICH analytical guidelines, indicating that the accuracy and precision of this qNMR method were qualified and suitable for quantitative analysis of *R. multiflora* samples. The ^1^H‐NMR spectra and data of the validation tests are presented in Figures S22–S33 and Table 1.

**TABLE 1:** The accuracy and precision data of the developed qNMR method for standard compounds.

Compound	HQC*^a^* (3.20 mg standard)		MQC*^a^* (0.80 mg standard)		LQC*^a^* (0.32 mg standard)	
	Intraday recovery ± SD (%)	Interday recovery ± SD (%)	Intraday recovery ± SD (%)	Interday recovery ± SD (%)	Intraday recovery ± SD (%)	Interday recovery ± SD (%)
THSG (**1**)	94.73 ± 1.36	94.69 ± 2.86	108.53 ± 0.96	104.88 ± 4.59	102.96 ± 5.13	100.00 ± 6.06
Emodin (**2**)	95.46 ± 2.12	94.14 ± 2.17	97.29 ± 4.44	96.30 ± 4.22	92.90 ± 0.88	96.88 ± 2.58
Physcion (**3**)	103.91 ± 2.66	102.50 ± 4.42	103.19 ± 1.77	103.70 ± 4.08	105.15 ± 0.97	106.25 ± 1.98
Rhein (**4**)	99.23 ± 2.66	99.38 ± 1.47	94.49 ± 1.85	94.12 ± 1.56	98.94 ± 7.63	94.12 ± 6.29

^*a*^HQC: high‐concentration group for quality control; MQC: medium‐concentration group for quality control; LQC: low‐concentration group for quality control.

### 4.4. Analysis of Raw and Commercial Processed Herbal Materials

This developed qNMR method was applied to analyze the commercial *R. multiflora* samples collected from the various local traditional Chinese medicine pharmaceutical suppliers. The relevant information of the samples is listed in Supporting Information (Table S1). Powdered *R. multiflora* samples were extracted and prepared according to the procedures described in the Experimental Part section, and the extraction results are listed in Table S2. The prepared samples were then dissolved in DMSO‐*d*_6_ for measurement by ^1^H‐NMR, and the resulting spectra are provided in Figures S34–S45. After ^1^H‐NMR analysis, the target peaks of THSG (**1**), emodin (**2**), physcion (**3**), rhein (**4**), and anthracene standard were selected to calculate the contents of these indicator compounds with the equation described above. The data for the contents of **1-4** in *R. multiflora* samples, meeting the specified criteria in the pharmacopeia, are presented in Table 2 and Table S4, respectively.

**TABLE 2:** The contents of THSG (**1**), emodin (**2**), physcion (**3**), and rhein (**4**) in *R. multiflora* herbal materials and decoction.

		Concentration ± SD (mM)				Contents ± SD (% in material)				
		THSG (1)	Emodin (2)	Physcion (3)	Rhein (4)	THSG (1)	Emodin (2)	Physcion (3)	Rhein (4)	Total contents of 2 and 3 (%)
Raw sample	1	4.54 ± 0.092	N.D.	0.23 ± 0.004	N.D.	2.94 ± 0.059	N.D.	0.05 ± 0.001	N.D.	0.05
	2	2.43 ± 0.144	N.D.	0.23 ± 0.023	N.D.	1.58 ± 0.093	N.D.	0.05 ± 0.005	N.D.	0.05
	3	2.74 ± 0.099	N.D.	0.38 ± 0.046	N.D.	1.77 ± 0.063	N.D.	0.09 ± 0.010	N.D.	0.09

Commercial‐processed sample	4	0.71 ± 0.006	N.D.	0.10 ± 0.020	N.D.	0.46 ± 0.004	N.D.	0.02 ± 0.004	N.D.	0.02
	5	1.07 ± 0.042	N.D.	0.04 ± 0.006	N.D.	0.69 ± 0.027	N.D.	0.01 ± 0.001	N.D.	0.01
	6	1.67 ± 0.026	0.27 ± 0.047	N.D.	N.D.	1.01 ± 0.017	0.06 ± 0.010	N.D.	N.D.	0.06

Microwave‐processed sample	7	1.72 ± 0.039	N.D.	N.D.	N.D.	1.12 ± 0.025	N.D.	N.D.	N.D.	N.D.
	8	1.49 ± 0.014	0.51 ± 0.012	0.19 ± 0.009	N.D.	0.96 ± 0.009	0.11 ± 0.002	0.04 ± 0.002	N.D.	0.15
	9	0.75 ± 0.022	0.54 ± 0.014	0.18 ± 0.021	N.D.	0.49 ± 0.014	0.12 ± 0.003	0.04 ± 0.005	N.D.	0.16
	10	0.69 ± 0.018	0.39 ± 0.011	0.09 ± 0.006	N.D.	0.45 ± 0.012	0.09 ± 0.002	0.02 ± 0.001	N.D.	0.11
	11	0.39 ± 0.004	N.D.	0.09 ± 0.10	N.D.	0.25 ± 0.002	N.D.	0.02 ± 0.002	N.D.	0.02

Decoction	12	0.53 ± 0.009	N.D.	N.D.	N.D.	0.34 ± 0.006	N.D.	N.D.	N.D.	N.D.
	13	0.53 ± 0.027	N.D.	N.D.	N.D.	0.34 ± 0.017	N.D.	N.D.	N.D.	N.D.
	14	0.85 ± 0.010	N.D.	N.D.	N.D.	0.30 ± 0.003	N.D.	N.D.	N.D.	N.D.

### 4.5. Analysis of the Microwave‐Processed Herbal Materials

In preliminary experiments performed in our laboratory, *Reynoutria multiflora* Radix samples MW1–3 were processed using a microwave oven with varying parameters (Table 3). The sample MW1 was the raw material of *Reynoutria multiflora* Radix. The power of sample MW3 was higher than that of sample MW2, but both were heated for the same amount of time during every processing step. Regarding the number of heating processing times, sample MW2 was heated for more times than sample MW3. After microwave processing, samples MW1–3 were analyzed for THSG (**1**) content by HPLC‐UV (Figure S60). Accordingly, samples MW1–3 contained 2.12%, 1.73%, and 0.62% of THSG (**1**), respectively. These results suggest that microwave processing can decrease the content of THSG (**1**) in *Reynoutria multiflora* Radix samples, thereby achieving the aim of traditional Chinese medicine processing. However, the content of THSG (**1**) in sample MW3 was below the limit of 0.7%, which was set in the criteria in the pharmacopeia, meaning that the power of the microwave for sample MW3 may be too high. Therefore, a series of protocols for the microwave processing of *Reynoutria multiflora* Radix samples referring to the conditions of sample MW2 are designed. The microwave‐processed samples 7–11 were derived from the raw sample 3, and the microwave processing parameters are listed in Table 4. The powders of processed samples as well as the commercial sample were prepared and examined. The ^1^H‐NMR spectra of microwave‐processed samples are provided in Figures S46–S53, and the contents of indicator compounds including THSG (**1**), emodin (**2**), physcion (**3**), and rhein (**4**) were calculated according to the equation. The afforded data of THSG (**1**), emodin (**2**), physcion (**3**), and rhein (**4**) in processed samples are shown in Table 2.

**TABLE 3:** The microwave‐processed conditions of *Reynoutria multiflora* radix samples MW1–3.

Sample	Power (Watt)	Duration (min)	Number of times
MW1	─	─	─
MW2	270	1	4
MW3	540	1	2

**TABLE 4:** The protocol of microwave processing for *Reynoutria multiflora* radix samples 7–11.

Sample	Power (Watt)	Duration (min)	Number of times
7	270	1	3
8	270	1	5
9	270	1	7
10	270	1	8
11	270	2	3

### 4.6. Analysis of Herbal Decoction

In clinical applications, *R. multiflora* was not often used as a single herb but showed better therapeutic effects when combined with other herbs in complex prescriptions. The common dosage forms of *R. multiflora* included Chinese patent drugs, powder, tea, and decoction for oral administration [15, 16]. The most famous one is Qi Bao Mei Ran Dan, a decoction with *R. multiflora* as its main ingredient. In Traditional Chinese Medicine, Qi Bao Mei Ran Dan is typically used to nourish the kidneys, replenish blood, and treat premature aging [11]. Several studies have explored the effects of Qi Bao Mei Ran Dan on the expression of apoptosis‐related genes and proteins in a human skin aging model, indicating that this decoction can improve the proliferative activity in aging cells and delay skin aging [21]. In this study, the qNMR method was applied to analyze the Qi Bao Mei Ran Dan decoction samples, among which *R. multiflora* was the major component and played a vital role in the pharmacological effect of this decoction. Although the commercial products of decoction from different suppliers have varying composition ratios (Table S5), the content of THSG (**1**) in *Reynoutria multiflora* Radix Preparata material can be calculated to evaluate the quality of the decoction products. The ^1^H‐NMR spectra of Qi Bao Mei Ran Dan decoction samples are provided in Figures S54–S59.

## 5. Discussion

According to the experimental results, the raw materials of samples 1–3 contained THSG (**1**) ranging from 1.58% to 2.94% of THSG (**1**). The contents of THSG (**1**) should be greater than 1.0% in the raw materials of *R. multiflora* specified in Taiwan Herbal Pharmacopeia (Table S4); thus, all the collected commercial samples conformed to the criteria [11]. The processed samples 4–6 exhibited significantly lower contents of THSG (**1**) than those in the raw samples, ranging from 0.46% to 1.01% in the processed samples. Based on previous reports related to Traditional Chinese Medicine, the chemical constituents, bioactivities, and toxicities of herbs vary depending on their processing. It had been reported that the contents of THSG (**1**) would decrease after processing [22], and this tendency was observed in our experimental results among both the raw and processed samples. Nevertheless, the commercial processed samples 4 and 5 displayed THSG (**1**) contents lower than 0.70%, which is the minimum limit of THSG (**1**) in the processed *R. multiflora* samples specified in the pharmacopeia of the People’s Republic of China (Table S4) [24]. It might indicate that the quality of these collected commercial *R. multiflora* samples would not meet the specified criteria. Regarding the contents of the anthraquinones, rhein (**4**) could not be detected in all the commercial samples in the ^1^H‐NMR spectra, supposing that rhein (**4**) existed in the *R. multiflora* samples below the LOD. The contents of emodin (**2**) in most commercial samples could not be estimated, except that Sample 6 presents the content of emodin (**2**) as 0.06%. All the commercial samples showed the presence of physcion (**3**) ranging from 0.01% to 0.09%, excluding Sample 6, in which physcion (**3**) was not detectable. As a summation of the contents of emodin (**2**) and physcion (**3**), all the commercial samples presented these two anthraquinones from 0.01% to 0.09%, among which were all below the minimum limit of 0.10% set in the pharmacopeia. It might indicate that the qualities of these collected commercial *Reynoutria multiflora* Radix samples would not meet the specified criteria.

In comparison with the results of raw samples, the contents of THSG (**1**) displayed a notable reduction in the microwave‐processed samples. The microwave‐processed samples contained 0.25%–1.12% of THSG (**1**) in the materials. Particularly, samples 7 and 8 exhibited the contents of THSG (**1**) as 1.12% and 0.96%, respectively, which comply with the criteria for processed *Reynoutria multiflora* Radix specified as 0.70% in the pharmacopeia. However, the contents of THSG (**1**) were below the minimum limit in samples 9–11. It suggested that longer duration or more times of microwave heating might result in the decomposition of THSG (**1**). Considering the results of anthraquinones in the microwave‐processed samples, rhein (**4**) could not be detected from all ^1^H‐NMR spectra because the contents of rhein (**4**) were lower than LOD. The exhibited contents of emodin (**2**) ranged from 0.09% to 0.12% in samples 8, 9, and 10, but emodin (**2**) was not detected in samples 7 and 11. Physcion (**3**) was detected in the microwave‐processed samples, ranging from 0.02% to 0.04%, except in sample 7. Regarding the total contents of emodin (**2**) and physcion (**3**), samples 8, 9, and 10 complied with the criteria of 0.10%, showing the range between 0.11% and 0.16%. Nevertheless, in samples 7 and 11, the sums of emodin (**2**) and physcion (**3**) were below the specification. Overall, the results of sample 8 exhibited acceptable contents of THSG (**1**) and anthraquinones, complying with the criteria specified in the pharmacopeia, indicating that its parameters were optimal for the protocols designed in this study. Comparatively, sample 7 exhibited a higher content of THSG (**1**) than that of sample 8, but undetectable anthraquinones. It meant that the number of microwave times in sample 7 might not satisfy the aims of traditional Chinese medicine processing. Although samples 9 and 10 showed the appropriate levels of anthraquinones, their contents of THSG (**1**) were out of specification. Due to the longer duration of microwave heating, sample 11 displayed the lowest contents of THSG (**1**) and anthraquinones among all microwave‐processed samples. Hence, the optimized microwave processing parameters of *Reynoutria multiflora* Radix should be based on the concept of shorter heating durations and a larger number of repetitions.

According to the qNMR analytical results of decoction samples, THSG (**1**) contents in samples 12–14 were all lower than the specified criteria of 0.70% in the pharmacopeia of the People’s Republic of China [23]. Regarding the contents of the free anthraquinones, the signals were not significant and covered by the sample matrix in Qi Bao Mei Ran Dan. Thus, the quantification of free anthraquinones in decoction samples is unachievable. Overall, the results showed that the contents of the indicator compounds in commercial decoction samples did not comply with the criteria for processed *R. multiflora* specified in the pharmacopeia. This may be due to the degradation or transformation of ingredients caused by heat during the manufacturing of commercial decoction products.

## 6. Conclusion

In this study, a ^1^H‐NMR method for the quantitative analysis of *R. multiflora* herbal materials and decoction has been developed. This quantitative method could be used to efficiently monitor the contents of THSG (**1**), emodin (**2**), physcion (**3**), and rhein (**4**), and the LODs and recovery percentages of indicator compounds were satisfactory in all the examined cases. According to the quantitative results of our study, the raw commercial samples exhibited higher contents of THSG (**1**) than the processed samples. However, the anthraquinones in some commercial samples were lower than the minimum limit specified in the pharmacopeia. In addition, microwave processed technology was applied to *Reynoutria multiflora* Radix, and the indicator compounds of the resulting products were examined by qNMR. A best‐fit microwave processing protocol and a modified ^1^H‐NMR method for quantitative analysis have been developed. Among the microwave‐processed samples, sample 11 displayed an acceptable result and compliance with the criteria specified in the pharmacopeia. The analysis of the decoction revealed that the manufacturing process may alter the composition of the ingredients in the herbal materials. As a result, the pharmacopoeia standards for the quality of decoctions may need to be re‐evaluated. In conclusion, the developed rapid and feasible ^1^H NMR quantitative method yields promising results without requiring any complicated pretreatments, such as chromatographic separation, derivatization, pH control, or the addition of NMR shifting reagents. Additionally, the analytical solvent system is both safe and convenient. Therefore, this method is applicable in the quantitative analysis of bioactive components in both raw and processed herbal materials, as well as in traditional herbal medicine decoctions.

## Author Contributions

Hao‐Ze Li: investigation, data curation, writing–original draft preparation, and writing–editing. Hong Nhung Phan: investigation, data curation, and writing–original draft preparation. Zih‐Hong Jhang: investigation and data curation. Guo‐Fen Chen: data curation (2D experiments). Mei‐Lin Yang: investigation and methodology. Tian‐Shung Wu: conceptualization, supervision, and writing–original draft preparation. Ping‐Chung Kuo: conceptualization, supervision, writing–original draft preparation, and writing–editing.

## Funding

The authors are grateful for financial support from the National Science and Technology Council (NSTC), Taiwan, awarded to Tian‐Shung Wu and Ping‐Chung Kuo. The authors also wish to thank Kaiser Pharmaceutical Co., Ltd., Tainan, Taiwan, for the partial financial support of the present research.

## Conflicts of Interest

The authors declare no conflicts of interest.

## Supporting Information

Additional supporting information can be found online in the Supporting Information section.

## Supporting information


**Supporting Information** Figures S1–S60 and Table S1–S5 could be found online in the Supporting Information file.

## Data Availability

The data that support the findings of this study are available from the corresponding author upon reasonable request.
