# Supplementary material for: A Feasible 1H‐NMR Quantification Method of Indicator Compounds in Microwave‐Processed Reynoutria multiflora Radix and Its Decoction
Source: Int J Anal Chem. 2026 Oct 18;2026:6092060. doi: 10.1155/ianc/6092060 (PMC13678417; doi:10.1155/ianc/6092060)
Supplement: Supplementary file 1 [file IANC-2026-6092060-s001.docx]

**Supporting Information**

**A Feasible ^1^H-NMR Quantification Method of Indicator Compounds in Microwave Processed *Reynoutria multiflora* Radix and its Decoction**

Hao-Ze Li^1,a^ ǀ Hong Nhung Phan^1,a^ ǀ Zih-Hong Jhang^1^ ǀ Guo-Fen Chen^2^ ǀ Mei-Lin Yang^1^ ǀ Tian-Shung Wu^1,^* ǀ and Ping-Chung Kuo^1,^*

^1^School of Pharmacy, College of Medicine, National Cheng Kung University, Tainan 70101, Taiwan ǀ ^2^Department of Chemistry, National Chung-Hsing University, Taichung 402, Taiwan

**Corresponding Authors**

Prof. Tian-Shung Wu.

E-mail address: [tswu@mail.ncku.edu.tw](mailto:tswu@mail.ncku.edu.tw)

Prof. Ping-Chung Kuo

E-mail address: [z10502016@ncku.edu.tw](mailto:z10502016@ncku.edu.tw)

**Contents**

**Figure S1.** The UPLC chromatograms of indicator compounds and *R. multiflora* sample.

**Figure S2.** ^1^H-NMR spectrum of THSG (**1**) (DMSO-*d_6_*, 400 MHz, 100 scans).

**Figure S3.** ^1^H-NMR spectrum of emodin (**2**) (DMSO-*d_6_*, 400 MHz, 100 scans).

**Figure S4.** ^1^H-NMR spectrum of physcion (**3**) (DMSO-*d_6_*, 400 MHz, 100 scans).

**Figure S5.** ^1^H-NMR spectrum of rhein (**4**) (DMSO-*d_6_*, 400 MHz, 100 scans).

**Figure S6.** ^1^H-NMR spectrum of THSG (**1**) LOD (DMSO-*d_6_*, 400 MHz, 100 scans).

**Figure S7.** Expanded 6-9 ppm region of ^1^H-NMR spectrum of THSG (**1**) LOD (DMSO-*d_6_*, 400 MHz, 100 scans).

**Figure S8.** ^1^H-NMR spectrum of THSG (**1**) LOQ (DMSO-*d_6_*, 400 MHz, 100 scans).

**Figure S9.** Expanded 6-9 ppm region of ^1^H-NMR spectrum of THSG (**1**) LOQ (DMSO-*d_6_*, 400 MHz, 100 scans).

**Figure S10.** ^1^H-NMR spectrum of emodin (**2**) LOD (DMSO-*d_6_*, 400 MHz, 100 scans).

**Figure S11.** Expanded 6-9 ppm region of ^1^H-NMR spectrum of emodin (**2**) LOD (DMSO-*d_6_*, 400 MHz, 100 scans).

**Figure S12**. ^1^H-NMR spectrum of emodin (**2**) LOQ (DMSO-*d_6_*, 400 MHz, 100 scans).

**Figure S13**. Expanded 6-9 ppm region of ^1^H-NMR spectrum of emodin (**2**) LOQ (DMSO-*d_6_*, 400 MHz, 100 scans).

**Figure S14.** ^1^H-NMR spectrum of physcion (**3**) LOD (DMSO-*d_6_*, 400 MHz, 100 scans).

**Figure S15.** Expanded 6-9 ppm region of ^1^H-NMR spectrum of physcion (**3**) LOD (DMSO-*d_6_*, 400 MHz, 100 scans).

**Figure S16.** ^1^H-NMR spectrum of physcion (**3**) LOQ (DMSO-*d_6_*, 400 MHz, 100 scans).

**Figure S17.** Expanded 6-9 ppm ^1^H-NMR spectrum of physcion (**3**) LOQ (DMSO-*d_6_*, 400 MHz, 100 scans).

**Figure S18.** ^1^H-NMR spectrum of rhein (**4**) LOD (DMSO-*d_6_*, 400 MHz, 100 scans).

**Figure S19.** Expanded 6-9 ppm region of ^1^H-NMR spectrum of rhein (**4**) LOD (DMSO-*d_6_*, 400 MHz, 100 scans).

**Figure S20.** ^1^H-NMR spectrum of rhein (**4**) LOQ (DMSO-*d_6_*, 400 MHz, 100 scans).

**Figure S21.** Expanded 7-8.5 ppm region of ^1^H-NMR spectrum of rhein (**4**) LOQ (DMSO-*d_6_*, 400 MHz, 100 scans).

**Figure S22.** ^1^H-NMR spectrum of THSG (**1**) HQC (DMSO-*d_6_*, 400 MHz, 100 scans, IS: anthracene).

**Figure S23.** ^1^H-NMR spectrum of THSG (**1**) MQC (DMSO-*d_6_*, 400 MHz, 100 scans, IS: anthracene).

**Figure S24.** ^1^H-NMR spectrum of THSG (**1**) LQC (DMSO-*d_6_*, 400 MHz, 100 scans, IS: anthracene).

**Figure S25.** ^1^H-NMR spectrum of emodin (**2**) HQC (DMSO-*d_6_*, 400 MHz, 100 scans, IS: anthracene).

**Figure S26.** ^1^H-NMR spectrum of emodin (**2**) MQC (DMSO-*d_6_*, 400 MHz, 100 scans, IS: anthracene).

**Figure S27.** ^1^H-NMR spectrum of emodin (**2**) LQC (DMSO-*d_6_*, 400 MHz, 100 scans, IS: anthracene).

**Figure S28.** ^1^H-NMR spectrum of physcion (**3**) HQC (DMSO-*d_6_*, 400 MHz, 100 scans, IS: anthracene).

**Figure S29.** ^1^H-NMR spectrum of physcion (**3**) MQC (DMSO-*d_6_*, 400 MHz, 100 scans, IS: anthracene).

**Figure S30.** ^1^H-NMR spectrum of physcion (**3**) LQC (DMSO-*d_6_*, 400 MHz, 100 scans, IS: anthracene).

**Figure S31.** ^1^H-NMR spectrum of rhein (**4**) HQC (DMSO-*d_6_*, 400 MHz, 100 scans, IS: anthracene).

**Figure S32.** ^1^H-NMR spectrum of rhein (**4**) MQC (DMSO-*d_6_*, 400 MHz, 100 scans, IS: anthracene).

**Figure S33.** ^1^H-NMR spectrum of rhein (**4**) LQC (DMSO-*d_6_*, 400 MHz, 100 scans, IS: anthracene).

**Figure S34.** ^1^H-NMR spectrum of solution A+IS of sample 1 (DMSO-*d_6_*, 400 MHz, 100 scans, IS: anthracene).

**Figure S35.** ^1^H-NMR spectrum of solution B+IS of sample 1 (DMSO-*d_6_*, 400 MHz, 100 scans, IS: anthracene).

**Figure S36.** ^1^H-NMR spectrum of solution A+IS of sample 2 (DMSO-*d_6_*, 400 MHz, 100 scans, IS: anthracene).

**Figure S37.** ^1^H-NMR spectrum of solution B+IS of sample 2 (DMSO-*d_6_*, 400 MHz, 100 scans, IS: anthracene).

**Figure S38.** ^1^H-NMR spectrum of solution A+IS of sample 3 (DMSO-*d_6_*, 400 MHz, 100 scans, IS: anthracene).

**Figure S39.** ^1^H-NMR spectrum of solution B+IS of sample 3 (DMSO-*d_6_*, 400 MHz, 100 scans, IS: anthracene).

**Figure S40.** ^1^H-NMR spectrum of solution A+IS of sample 4 (DMSO-*d_6_*, 400 MHz, 100 scans, IS: anthracene).

**Figure S41.** ^1^H-NMR spectrum of solution B+IS of sample 4 (DMSO-*d_6_*, 400 MHz, 100 scans, IS: anthracene).

**Figure S42.** ^1^H-NMR spectrum of solution A+IS of sample 5 (DMSO-*d_6_*, 400 MHz, 100 scans, IS: anthracene).

**Figure S43.** ^1^H-NMR spectrum of solution B+IS of sample 5 (DMSO-*d_6_*, 400 MHz, 100 scans, IS: anthracene).

**Figure S44.** ^1^H-NMR spectrum of solution A+IS of sample 6 (DMSO-*d_6_*, 400 MHz, 100 scans, IS: anthracene).

**Figure S45.** ^1^H-NMR spectrum of solution B+IS of sample 6 (DMSO-*d_6_*, 400 MHz, 100 scans, IS: anthracene).

**Figure S46.** ^1^H-NMR spectrum of solution A+IS of sample 7 (DMSO-*d_6_*, 400 MHz, 100 scans, IS: anthracene).

**Figure S47.** ^1^H-NMR spectrum of solution B+IS of sample 7 (DMSO-*d_6_*, 400 MHz, 100 scans, IS: anthracene).

**Figure S48.** ^1^H-NMR spectrum of solution A+IS of sample 9 (DMSO-*d_6_*, 400 MHz, 100 scans, IS: anthracene).

**Figure S49.** ^1^H-NMR spectrum of solution B+IS of sample 9 (DMSO-*d_6_*, 400 MHz, 100 scans, IS: anthracene).

**Figure S50.** ^1^H-NMR spectrum of solution A+IS of sample 10 (DMSO-*d_6_*, 400 MHz, 100 scans, IS: anthracene).

**Figure S51.** ^1^H-NMR spectrum of solution B+IS of sample 10 (DMSO-*d_6_*, 400 MHz, 100 scans, IS: anthracene).

**Figure S52.** ^1^H-NMR spectrum of solution A+IS of sample 11 (DMSO-*d_6_*, 400 MHz, 100 scans, IS: anthracene).

**Figure S53.** ^1^H-NMR spectrum of solution B+IS of sample 11 (DMSO-*d_6_*, 400 MHz, 100 scans, IS: anthracene).

**Figure S54.** ^1^H-NMR spectrum of solution A+IS of sample 12 (DMSO-*d_6_*, 400 MHz, 100 scans, IS: anthracene).

**Figure S55.** ^1^H-NMR spectrum of solution B+IS of sample 12 (DMSO-*d_6_*, 400 MHz, 100 scans, IS: anthracene).

**Figure S56.** ^1^H-NMR spectrum of solution A+IS of sample 13 (DMSO-*d_6_*, 400 MHz, 100 scans, IS: anthracene).

**Figure S57.** ^1^H-NMR spectrum of solution B+IS of sample 13 (DMSO-*d_6_*, 400 MHz, 100 scans, IS: anthracene).

**Figure S58.** ^1^H-NMR spectrum of solution A+IS of sample 14 (DMSO-*d_6_*, 400 MHz, 100 scans, IS: anthracene).

**Figure S59.** ^1^H-NMR spectrum of solution B+IS of sample 14 (DMSO-*d_6_*, 400 MHz, 100 scans, IS: anthracene).

**Figure S60.** The chromatograms of (A) THSG, (B) MW1, (C) MW2, (D) MW3.

**Table S1.** Information on Samples 1-14 analyzed in this study.

**Table S2.** Extraction and preparation of *R. multiflora* samples for qNMR analysis.

**Table S3.** The acquisition parameters for 2D NMR experiments.

**Table S4.** The criteria of indicated compounds in pharmacopoeia in various countries.

**Table S5.** Composition ratio of Qi Bao Mei Ran Dan decoction samples 12-14.

(A)

(B)

(C)

(D)

(E)

(F)

**Figure S1.** The UPLC chromatograms of indicator compounds and *R. multiflora* sample. (A) and (B) are chromatograms of THSG at 280 and 320 nm; (C) and (D) are chromatograms of mixed standards of THSG, emodin and physcion at 254 and 320 nm; and (E) and (F) are chromatograms of *R. multiflora* sample at 280 and 320 nm.

**Figure S2.** ^1^H-NMR spectrum of THSG (**1**) (DMSO-*d_6_*, 400 MHz, 100 scans).

THSG, ^1^H-NMR (DMSO-*d*_6_, 400 MHz) δ: 3.24 (1H, m, H-6'') 3.25 (1H, m, H-3''), 3.26 (1H, m, H-4''), 3.29 (1H, m, H-5''), 3.67 (1H, m, H-2''), 4.40 (1H, d, *J* = 8.0 Hz, H-1''), 6.17 (1H, d, *J* = 2.8 Hz, H-4), 6.54 (1H, d, *J* = 2.8 Hz, H-6), 6.73 (2H, d, *J* = 8.0 Hz, H-3', 5'), 6.88 (1H, d, *J* = 16.4 Hz, H-8), 7.43 (2H, d, *J* = 8.0 Hz, H-2', 6'), 7.67 (1H, d, *J* = 16.4 Hz, H-7).

**Figure S3.** ^1^H-NMR spectrum of emodin (**2**) (DMSO-*d_6_*, 400 MHz, 100 scans).

Emodin, ^1^H-NMR (DMSO-*d*_6_, 400 MHz) δ: 2.43 (3H, s, CH_3_-6), 6.60 (1H, d, *J* = 2.4 Hz, H-2), 7.14 (1H, d, *J* = 2.4 Hz, H-4), 7.20 (1H, d, *J* = 2.4 Hz, H-7), 7.53 (1H, d, *J* = 1.6 Hz, H-5), 12.07 (1H, s, OH-8), 12.12 (1H, s, OH-1).

**Figure S4.** ^1^H-NMR spectrum of physcion (**3**) (DMSO-*d_6_*, 400 MHz, 100 scans).

Physcion, ^1^H-NMR (DMSO-*d*_6_, 400 MHz) δ: 2.43 (3H, s, CH_3_-6), 3.94 (3H, s, OCH_3_-3), 6.90 (1H, d, *J* = 2.4 Hz, H-2), 7.22 (2H, m, H-4, 7), 7.55 (1H, d, *J* = 1.6 Hz, H-5), 11.99 (1H, s, OH-1), 12.20 (1H, s, OH-8).

**Figure S5.** ^1^H-NMR spectrum of rhein (**4**) (DMSO-*d_6_*, 400 MHz, 100 scans).

Rhein, ^1^H-NMR (DMSO-*d*_6_, 400 MHz) δ: 7.43 (1H, dd, *J* = 8.3, 1.1 Hz, H-7), 7.76 (1H, dd, *J* = 7.6, 1.2 Hz, H-5), 7.79 (1H, d, *J* = 1.2 Hz, H-2), 7.85 (1H, dd, *J* = 7.6, 7.6 Hz, H-6), 8.16 (1H, d, *J* = 1.6 Hz, H-4), 11.93 (2H, s, OH-1, 8)

**Figure S6.** ^1^H-NMR spectrum of THSG (**1**) LOD (DMSO-*d_6_*, 400 MHz, 100 scans).

**Figure S7.** Expanded 6-9 ppm region of ^1^H-NMR spectrum of THSG (**1**) LOD (DMSO-*d_6_*, 400 MHz, 100 scans).

**Figure S8.** ^1^H-NMR spectrum of THSG (**1**) LOQ (DMSO-*d_6_*, 400 MHz, 100 scans).

**Figure S9.** Expanded 6-9 ppm region of ^1^H-NMR spectrum of THSG (**1**) LOQ (DMSO-*d_6_*, 400 MHz, 100 scans).

**Figure S10**. ^1^H-NMR spectrum of emodin (**2**) LOD (DMSO-*d_6_*, 400 MHz, 100 scans).

**Figure S11.** Expanded 6-9 ppm region of ^1^H-NMR spectrum of emodin (**2**) LOD (DMSO-*d_6_*, 400 MHz, 100 scans).

**Figure S12**. ^1^H-NMR spectrum of emodin (**2**) LOQ (DMSO-*d_6_*, 400 MHz, 100 scans).

**Figure S13**. Expanded 6-9 ppm region of ^1^H-NMR spectrum of emodin (**2**) LOQ (DMSO-*d_6_*, 400 MHz, 100 scans).

**Figure S14.** ^1^H-NMR spectrum of physcion (**3**) LOD (DMSO-*d_6_*, 400 MHz, 100 scans).

**Figure S15.** Expanded 6-9 ppm region of ^1^H-NMR spectrum of physcion (**3**) LOD (DMSO-*d_6_*, 400 MHz, 100 scans).

**Figure S16.** ^1^H-NMR spectrum of physcion (**3**) LOQ (DMSO-*d_6_*, 400 MHz, 100 scans).

**Figure S17.** Expanded 6-9 ppm ^1^H-NMR spectrum of physcion (**3**) LOQ (DMSO-*d_6_*, 400 MHz, 100 scans).

**Figure S18.** ^1^H-NMR spectrum of rhein (**4**) LOD (DMSO-*d_6_*, 400 MHz, 100 scans).

**Figure S19.** Expanded 7-8.5 ppm region of ^1^H-NMR spectrum of rhein (**4**) LOD (DMSO-*d_6_*, 400 MHz, 100 scans).

**Figure S20.** ^1^H-NMR spectrum of rhein (**4**) LOQ (DMSO-*d_6_*, 400 MHz, 100 scans).

**Figure S21.** Expanded 7-8.5 ppm region of ^1^H-NMR spectrum of rhein (**4**) LOQ (DMSO-*d_6_*, 400 MHz, 100 scans).

**Figure S22.** ^1^H-NMR spectrum of THSG (**1**) HQC (DMSO-*d_6_*, 400 MHz, 100 scans, IS: anthracene).

**Figure S23.** ^1^H-NMR spectrum of THSG (**1**) MQC (DMSO-*d_6_*, 400 MHz, 100 scans, IS: anthracene).

**Figure S24.** ^1^H-NMR spectrum of THSG (**1**) LQC (DMSO-*d_6_*, 400 MHz, 100 scans, IS: anthracene).

**Figure S25.** ^1^H-NMR spectrum of emodin (**2**) HQC (DMSO-*d_6_*, 400 MHz, 100 scans, IS: anthracene).

**Figure S26.** ^1^H-NMR spectrum of emodin (**2**) MQC (DMSO-*d_6_*, 400 MHz, 100 scans, IS: anthracene).

**Figure S27.** ^1^H-NMR spectrum of emodin (**2**) LQC (DMSO-*d_6_*, 400 MHz, 100 scans, IS: anthracene).

**Figure S28.** ^1^H-NMR spectrum of physcion (**3**) HQC (DMSO-*d_6_*, 400 MHz, 100 scans, IS: anthracene).

**Figure S29.** ^1^H-NMR spectrum of physcion (**3**) MQC (DMSO-*d_6_*, 400 MHz, 100 scans, IS:anthracene).

**Figure S30.** ^1^H-NMR spectrum of physcion (**3**) LQC (DMSO-*d_6_*, 400 MHz, 100 scans, IS: anthracene).

**Figure S31.** ^1^H-NMR spectrum of rhein (**4)** HQC (DMSO-*d_6_*, 400 MHz, 100 scans, IS: anthracene).

**Figure S32.** ^1^H-NMR spectrum of rhein (**4)** MQC (DMSO-*d_6_*, 400 MHz, 100 scans, IS: anthracene).

**Figure S33.** ^1^H-NMR spectrum of rhein (**4)** LQC (DMSO-*d_6_*, 400 MHz, 100 scans, IS: anthracene).

**Figure S34.** ^1^H-NMR spectrum of solution A+IS of sample 1 (DMSO-*d_6_*, 400 MHz, 100 scans, IS: anthracene).

**Figure S35.** ^1^H-NMR spectrum of solution B+IS of sample 1 (DMSO-*d_6_*, 400 MHz, 100 scans, IS: anthracene).

**Figure S36.** ^1^H-NMR spectrum of solution A+IS of sample 2 (DMSO-*d_6_*, 400 MHz, 100 scans, IS: anthracene).

**Figure S37.** ^1^H-NMR spectrum of solution B+IS of sample 2 (DMSO-*d_6_*, 400 MHz, 100 scans, IS: anthracene).

**Figure S38.** ^1^H-NMR spectrum of solution A+IS of sample 3 (DMSO-*d_6_*, 400 MHz, 100 scans, IS: anthracene).

**Figure S39.** ^1^H-NMR spectrum of solution B+IS of sample 3 (DMSO-*d_6_*, 400 MHz, 100 scans, IS: anthracene).

**Figure S40.** ^1^H-NMR spectrum of solution A+IS of sample 4 (DMSO-*d_6_*, 400 MHz, 100 scans, IS: anthracene).

**Figure S41.** ^1^H-NMR spectrum of solution B+IS of sample 4 (DMSO-*d_6_*, 400 MHz, 100 scans, IS: anthracene).

**Figure S42.** ^1^H-NMR spectrum of solution A+IS of sample 5 (DMSO-*d_6_*, 400 MHz, 100 scans, IS: anthracene).

**Figure S43.** ^1^H-NMR spectrum of solution B+IS of sample 5 (DMSO-*d_6_*, 400 MHz, 100 scans, IS: anthracene).

**Figure S44.** ^1^H-NMR spectrum of solution A+IS of sample 6 (DMSO-*d_6_*, 400 MHz, 100 scans, IS: anthracene).

**Figure S45.** ^1^H-NMR spectrum of solution B+IS of sample 6 (DMSO-*d_6_*, 400 MHz, 100 scans, IS: anthracene).

**Figure S46.** ^1^H-NMR spectrum of solution A+IS of sample 7 (DMSO-*d_6_*, 400 MHz, 100 scans, IS: anthracene).

**Figure S47.** ^1^H-NMR spectrum of solution B+IS of sample 7 (DMSO-*d_6_*, 400 MHz, 100 scans, IS: anthracene).

**Figure S48.** ^1^H-NMR spectrum of solution A+IS of sample 9 (DMSO-*d_6_*, 400 MHz, 100 scans, IS: anthracene).

**Figure S49.** ^1^H-NMR spectrum of solution B+IS of sample 9 (DMSO-*d_6_*, 400 MHz, 100 scans, IS: anthracene).

**Figure S50.** ^1^H-NMR spectrum of solution A+IS of sample 10 (DMSO-*d_6_*, 400 MHz, 100 scans, IS: anthracene).

**Figure S51.** ^1^H-NMR spectrum of solution B+IS of sample 10 (DMSO-*d_6_*, 400 MHz, 100 scans, IS: anthracene).

**Figure S52.** ^1^H-NMR spectrum of solution A+IS of sample 11 (DMSO-*d_6_*, 400 MHz, 100 scans, IS: anthracene).

**Figure S53.** ^1^H-NMR spectrum of solution B+IS of sample 11 (DMSO-*d_6_*, 400 MHz, 100 scans, IS: anthracene).

**Figure S54.** ^1^H-NMR spectrum of solution A+IS of sample 12 (DMSO-*d_6_*, 400 MHz, 100 scans, IS: anthracene).

**Figure S55.** ^1^H-NMR spectrum of solution B+IS of sample 12 (DMSO-*d_6_*, 400 MHz, 100 scans, IS: anthracene).

**Figure S56.** ^1^H-NMR spectrum of solution A+IS of sample 13 (DMSO-*d_6_*, 400 MHz, 100 scans, IS: anthracene).

**Figure S57.** ^1^H-NMR spectrum of solution B+IS of sample 13 (DMSO-*d_6_*, 400 MHz, 100 scans, IS: anthracene).

**Figure S58.** ^1^H-NMR spectrum of solution A+IS of sample 14 (DMSO-*d_6_*, 400 MHz, 100 scans, IS: anthracene).

**Figure S59.** ^1^H-NMR spectrum of solution B+IS of sample 14 (DMSO-*d_6_*, 400 MHz, 100 scans, IS: anthracene).

(A)

(B)

(C)

(D)

**Figure S60.** The chromatograms of (A) THSG, (B) MW1, (C) MW2, (D) MW3.

**Table S1.** Information on Samples 1-14 analyzed in this study.

| **Sample** | **Type** | **Dosage form** | **Authenticated by** *^a^* |
| --- | --- | --- | --- |
| 1 | Raw material | slices | Kaiser Pharmaceutical Co. |
| 2 | Raw material | slices | Yong Xing Chinese Medicine Co. |
| 3 | Raw material | slices | Chuang Song Zong Pharmaceutical Co., Ltd. |
| 4 | Commercial-processed | slices | Chuang Song Zong Pharmaceutical Co., Ltd. |
| 5 | Commercial-processed | slices | Yong Xing Chinese Medicine Co |
| 6 | Commercial-processed | slices | Chia Jengs Enterprise Co., Ltd. |
| 7 | Microwave-processed | slices |  |
| 8 | Microwave-processed | slices |  |
| 9 | Microwave-processed | slices |  |
| 10 | Microwave-processed | slices |  |
| 11 | Microwave-processed | slices |  |
| 12 | Decoction | Powder | Chuang Song Zong Pharmaceutical Co., Ltd. |
| 13 | Decoction | Powder | Kaiser Pharmaceutical Co. |
| 14 | Decoction | Powder | Sheng Chang Pharmaceutical Company. |

*^a^* The herbal materials and decoctions used in this study were purchased by our research institute and authenticated by

qualified traditional Chinese medicine manufacturers in Taiwan. Samples 7–11 were prepared in our laboratory by subjecting Sample 3 to microwave processing under different conditions.

**Table S2.** Extraction and preparation of *R. multiflora* samples for qNMR analysis.

| **Sample** | **Powder (g)** | **Extract (mg)** | **Water layer** | **Chloroform layer** | **Internal standard**  **(mg)** |
| --- | --- | --- | --- | --- | --- |
|  |  |  | **Dilute ratio** | **Dilute ratio** |  |
| 1 | 0.5024 | 96.64 | 1:10 | 1:5 | 0.51 |
| 2 | 0.5017 | 64.42 | 1:10 | 1:5 | 0.51 |
| 3 | 0.5010 | 107.30 | 1:10 | 1:5 | 0.50 |
| 4 | 0.5025 | 25.70 | 1:10 | 1:5 | 0.53 |
| 5 | 0.5019 | 93.74 | 1:10 | 1:5 | 0.51 |
| 6 | 0.5025 | 126.20 | 1:10 | 1:5 | 0.50 |
| 7 | 0.5019 | 84.95 | 1:10 | 1:5 | 0.53 |
| 8 | 0.5017 | 31.27 | 1:10 | 1:5 | 0.50 |
| 9 | 0.5021 | 26.26 | 1:10 | 1:5 | 0.50 |
| 10 | 0.5025 | 18.92 | 1:10 | 1:5 | 0.51 |
| 11 | 0.5023 | 41.11 | 1:10 | 1:5 | 0.50 |
| 12 | 0.6017 | 173.22 | 1:10 | 1:5 | 0.52 |
| 13 | 0.6030 | 129.80 | 1:10 | 1:5 | 0.53 |
| 14 | 0.6904 | 210.99 | 1:10 | 1:5 | 0.53 |

**Table S3.** The acquisition parameters for 2D NMR experiments.

| **gHSQCAD** | **gHMBCAD** | **gHSQCADTOCSY** |
| --- | --- | --- |
| relaxation delay: 1s | relaxation delay: 1s | relaxation delay: 1s |
| steady-state: 32 | steady-state: 32 | steady-state: 32 |
| number of points: 1442 | number of points: 1442 | number of points: 1442 |
| increment: 96 | increment: 200 | increment: 128 |
| NUS rate: 25% | NUS rate: 25% | NUS rate: 25% |
| Scan number: 32 | Scan number: 64 | Scan number: 32 |
| *^1^J* coupling: 146Hz | *^1^J* suppression coupling:  165 to 130Hz | *^1^J* coupling: 146Hz |
|  | *^n^J* coupling: 8Hz (transfer delay is 62.5ms) | Spinlock duration:  100 ms |

**Table S4.** The criteria of indicated compounds in pharmacopoeia in various countries.

| **Pharmacopoeia** | ***Reynoutria multiflora* Radix** | ***Reynoutria multiflora* Radix Preparata** |
| --- | --- | --- |
| Taiwan Herbal Pharmacopeia 4^rd^ Edition | - THSG ≥ 1.0 % - Ethanol extractives ≥ 20.0 % - Water extractives ≥ 20.0 % | ─ |
| Pharmacopeia of the People’s Republic of China 2020 edition | - THSG ≥ 1.0 % - Emodin + Physcion ≥ 0.10 % (combined anthraquinones) - Water content ≤ 10.0 % | - THSG ≥ 0.70 % - Emodin + Physcion ≥ 0.10 % (free anthraquinones) - Water content ≤ 12.0 % |
| Hong Kong Chinese materia medica standards | - THSG ≥ 2.2 % - Water content ≤ 12.0 % | ─ |
| Korean Pharmacopeia 12^th^ revision | - THSG ≥ 0.75 % - Emodin + Physcion ≥ 0.10 % | ─ |
| The Japanese Pharmacopoeia *VXIII* | - Ethanol extractives ≥ 17.0 % | ─ |

**Table S5.** Composition ratio of Qi Bao Mei Ran Dan decoction samples 12-14.

| **Sample** | ***Reynoutria multiflora* Radix Preparata Contents (g/g material)** |
| --- | --- |
| 12 | 0.83 |
| 13 | 0.83 |
| 14 | 1.33 |
